# Differential effects of soluble and plaque amyloid on late-life depression: The moderating role of tau pathology

**DOI:** 10.1016/j.tjpad.2025.100318

**Published:** 2025-08-05

**Authors:** Gihwan Byeon, Suhyung Kim, Sunghwan Kim, Yoo Hyun Um, Sheng-Min Wang, Seunggyun Ha, Sonya Youngju Park, Yeong Sim Choe, Donghyeon Kim, Hyun Kook Lim, Chang Uk Lee, Dong Woo Kang

**Affiliations:** a Department of Psychiatry, Seoul St. Mary’s Hospital, College of Medicine, The Catholic University of Korea, 222 Banpo-daero, Seocho-gu, Seoul 06591, Republic of Korea; b Department of Psychiatry, St. Vincent’s Hospital, College of Medicine, The Catholic University of Korea, 93 Jungbu-daero, Paldal-gu, Suwon-si, Gyeonggi-do 16247, Republic of Korea; c Department of Psychiatry, Yeouido St. Mary’s Hospital, College of Medicine, The Catholic University of Korea, 10 63-ro, Yeongdeungpo-gu, Seoul 07345, Republic of Korea; d Division of Nuclear Medicine, department of Radiology, Seoul St. Mary's Hospital, College of Medicine, The Catholic University of Korea, 222 Banpo-daero, Seocho-gu, Seoul 06591, Republic of Korea; e Division of Nuclear Medicine, Yeouido St. Mary’s Hospital, College of Medicine, The Catholic University of Korea, 10 63-ro, Yeongdeungpo-gu, Seoul 07345, Republic of Korea; f Research Institute, NEUROPHET Inc., 5F, 27 Teheran-ro 4-gil, Gangnam-gu, Seoul 06232, Republic of Korea; g CMC Institute for Basic Medical Science, The Catholic Medical Center of The Catholic University of Korea, 222 Banpo-daero, Seocho-gu, Seoul 06591, Republic of Korea

**Keywords:** Alzheimer disease, Amyloid beta-peptides, Tau proteins, Depression

## Abstract

**Background:**

Late-life depression frequently co-occurs with Alzheimer’s disease (AD); however, the interactive effects of amyloid-beta (Aβ) species and tau pathology on depressive symptoms remain unclear. Soluble oligomeric Aβ (OAβ) and amyloid plaques may differentially influence depression depending on tau burden.

**Objectives:**

To examine how plasma OAβ and PET-measured amyloid plaque burden are associated with depressive symptoms across varying levels of tau pathology.

**Design:**

Cross-sectional analysis using generalized linear models with interaction terms, supported by stratified subgroup analyses and Johnson–Neyman procedures.

**Setting:**

Memory disorder clinic at a university-affiliated hospital.

**Participants:**

A total of 103 individuals, including cognitively normal controls (*n* = 24), patients with mild cognitive impairment (*n* = 54), and amyloid-positive dementia (*n* = 25), all of whom underwent plasma biomarker testing and tau and amyloid PET imaging.

**Measurements:**

Depression was evaluated using the Cornell Scale for Depression in Dementia (CSDD), Hamilton Depression Rating Scale (HAM-D), and Geriatric Depression Scale–Short Version (GDS-SV). Plasma OAβ was measured by Multimer Detection System (MDS), and PET quantified amyloid and tau burden.

**Results:**

MDS-OAβ showed a significant negative interaction with tau PET SUVR on depression scores (FDR-adjusted *p* < 0.05). Higher OAβ levels were linked to greater depression severity in low-tau individuals, but inversely related in high-tau individuals. Amyloid plaque burden was associated with depression only in those with advanced tau pathology.

**Conclusions:**

The association between amyloid pathology and depression differs depending on tau burden. Soluble OAβ may be a key contributor to depressive symptoms in early AD stages, while plaque effects become prominent later. These findings underscore the potential utility of OAβ as an early neuropsychiatric biomarker in AD and highlight the need to consider tau pathology when evaluating amyloid-related mood disturbances.

## 1 Introduction

Alzheimer’s disease (AD) is a neurodegenerative disorder that primarily affects the older adults, with a globally increasing prevalence [1]. Depression is also a common psychiatric illness in late life, affecting approximately 3 % of the healthy older adult population. However, its prevalence rises significantly among medically ill, hospitalized, or institutionalized older individuals. In particular, the prevalence of depression in patients with AD is notably high, ranging from 20 to 30 % [2,3]. Furthermore, late-life depression has been identified as a potential predictor of subsequent development of AD [4,5]. These findings suggest a close relationship between the two conditions.

Previous studies have shown that the major pathological hallmarks of AD, amyloid-beta (Aβ) and tau [6], are also associated with depression. A systematic review reported that non-demented older adults with depressive symptoms had significantly higher levels of Aβ than those without such symptoms [7], On the other hand, cognitively normal older adults with depressive symptoms exhibited a higher frequency of amyloid positivity, whereas those with mild cognitive impairment (MCI) showed the opposite trend [8].

Regarding tau pathology, a meta-analysis found no significant difference in tau burden between individuals with major depressive disorder (MDD) and those without [9]. In contrast, an imaging study demonstrated a significant association between depressive symptoms and tau deposition in the inferior temporal lobe and entorhinal cortex among cognitively normal older adults, suggesting the possibility of region-specific effects [10]. Similarly, another study found that tau deposition, but not amyloid deposition, was significantly associated with a diagnosis of depression in cognitively normal individuals [11].

Despite these findings, the interaction between Aβ and tau pathology in relation to depressive symptoms remains poorly understood. Given that both Aβ and tau are core biomarkers of AD, examining their combined effects is essential to understanding the full pathological basis of depression in the context of AD. Furthermore, the synergistic interaction between Aβ and tau pathology has been shown to play a critical role in the progression of AD [12], underscoring the need to consider their interaction when evaluating their association with depressive symptoms.

Moreover, there has been a lack of research examining the differential impact of specific pathologic amyloid species on depression. Among these, soluble oligomerized Aβ (OAβ) has been identified as the most neurotoxic species, primarily due to its ability to disrupt synaptic function, induce neuroinflammation, and impair neuronal viability even in the absence of plaque formation [13]. The recent development of multimer detection system (MDS) technology has improved the accuracy of detecting OAβ [14]. Distinguishing MDS-measured OAβ from amyloid plaque as measured by positron emission tomography (PET) imaging is essential to evaluating the predictive value of Aβ pathology in depression.

Therefore, in the present study, we aimed to investigate how MDS-measured OAβ and amyloid PET-detected Aβ pathology interact with tau pathology, as assessed by tau PET imaging, in relation to depressive symptoms. To capture depressive symptoms across a broad cognitive spectrum, we employed a multimodal assessment approach incorporating both clinician-rated and self-reported measures, thereby enabling a comprehensive evaluation of late-life depression in individuals with varying cognitive status.

## 2 Methods

### 2.1 Participants

A total of 373 participants were initially enrolled in the study, comprising 106 cognitively normal older adults (NC) with negative Aβ-PET scans, 18 Aβ-PET-positive NC, 116 Aβ-PET-negative patients with MCI, 101 Aβ-PET-positive MCI patients, and 32 Aβ-PET-positive dementia patients. These individuals were recruited from the Catholic Aging Brain Imaging database (CABID), which includes MRI and PET scans and clinical data of the older adults who visited the outpatient clinic at the Catholic Brain Health Center, Yeouido St. Mary’s Hospital, the Catholic University of Korea, from 2018 to 2024 **(Supplementary Fig. 1).**

The inclusion criteria were as follows: (1) age ≥ 55 years and (2) absence of clinically significant psychiatric disorders, such as MDD, schizophrenia, or bipolar disorder. The cognitive normality of NC was confirmed using the Korean version of the Consortium to Establish a Registry for Alzheimer's Disease (CERAD-K) battery [15], with a global Clinical Dementia Rating (CDR) score of 0. MCI was diagnosed based on the following criteria: (1) subjective memory complaints corroborated by an informant, (2) objective cognitive impairment in at least one CERAD-K domain (≥1.0 SD below age- and education-adjusted norms), (3) preserved activities of daily living, (4) a global CDR score of 0.5, and (5) absence of dementia according to DSM-V criteria. Patients with dementia due to AD met the criteria for probable AD as defined by Neurological and Communicative Disorders and Stroke and AD and Related Disorders Association (NINCDS-ADRDA) and DSM-V, in addition to having Aβ-PET-positive findings [16,17], with a global CDR score of 1. Exclusion criteria included systemic diseases known to cause cognitive impairment, severe sensory impairments, neurological conditions (e.g., brain tumors, epilepsy), cerebrovascular disease, or contraindications for imaging. All diagnoses were confirmed by two psychiatric specialists.

From the initial cohort of 373 subjects, a subset of 103 participants (24 NC, 54 MCI, and 25 Aβ-PET-positive dementia patients) with available depression scores, as well as Tau-PET scans, were selected for further analysis **(Supplementary Fig. 1).** All assessments, including brain imaging, blood sampling, and cognitive evaluations, were conducted within a three-month period at baseline. The study was conducted in accordance with ethical guidelines and approved by the Institutional Review Board (IRB No.: SC21TISI0017). Written informed consent was obtained from all participants.

### 2.2 Measurement of Aβ oligomerization in plasma

Plasma levels of OAβ were quantified using the Multimer Detection System for OAβ (MDS-OAβ). Blood samples were collected via venipuncture using ethylene-diamine-tetraacetic acid (EDTA) vacutainer tubes, following established protocols for MDS-OAβ measurement [18]. After collection, EDTA plasma was centrifuged at 3500 rpm for 15 min at room temperature. The resulting plasma was aliquoted into 1.5-mL polypropylene tubes and stored at −70 °C to −80 °C. The samples were then transported to PeopleBio Inc. for MDS-OAβ level analysis.

Prior to measurement, plasma aliquots were thawed at 37 °C for 15 min. MDS-OAβ levels were measured using the multimer detection system, which is CE-marked and approved by the Korean Food and Drug Administration [[18], [19], [20]]. This plasma-based biomarker has been previously validated against amyloid PET positivity, with a proposed cutoff value of 0.78 ng/mL for identifying Aβ positivity [21]. Furthermore, to classify AD risk, a two-cutoff algorithm was implemented, aligning with methodologies outlined in prior biomarker research [22]. Based on this approach, plasma MDS-OAβ levels were categorized into three risk groups: low risk (<0.78 ng/mL), intermediate risk (0.78–0.93 ng/mL), and high risk (≥0.93 ng/mL). The cutoff values were determined based on the CLSI guideline NBS 04-A, which outlines standardized procedures for establishing and validating reference intervals in clinical laboratory testing.

### 2.3 Image acquisition

All participants underwent neuroimaging using a Siemens Skyra 3T MRI scanner equipped with a 20-channel head and neck coil (Siemens Healthcare, Erlangen, Germany) for high-resolution 3D T1-weighted structural imaging. In addition, PET scans were performed using a Biograph 40 TruePoint system (Siemens Medical Solutions, Erlangen, Germany) with radiotracers [¹⁸F]-flutemetamol and [¹⁸F]-flortaucipir. PET image acquisition was initiated 90 min following intravenous administration of 185 MBq [¹⁸F]-flutemetamol, or 80 min after administration of 370 MBq [¹⁸F]-flortaucipir, with static scans acquired over a 20-minute duration. Images were reconstructed using a 2D ordered-subsets expectation-maximization (OSEM) algorithm with two iterations and 21 subsets. The final image matrix measured 256 × 256 × 175, with a voxel resolution of 1.3364 × 1.3364 × 3 mm³. Prior to PET acquisition, low-dose CT scans were obtained for attenuation correction. T1-weighted MRI scans were acquired using a magnetization-prepared rapid gradient echo (MPRAGE) sequence with the following parameters: TR = 1860 ms, TE = 25.3 ms, flip angle = 9°, FOV = 224 × 224 mm, matrix size = 256 × 256, 208 slices, and a 1.0 mm slice thickness. All imaging data were anonymized and converted from DICOM to NIfTI format using the dcm2niix conversion tool.

### 2.4 Image preprocessing

SCALE PET 2.0 (Neurophet Inc., Seoul, Republic of Korea) was used for imaging preprocessing and quantification of [^18^F]-flutemetamol and [^18^F]-flortaucipir PET/CT scans [[23], [24], [25]]. This process involved brain parcellation on coregistered T1-weighted MRI scans. T1-weighted MRIs were corrected for non-uniformity and field distortions before processing. PET images were registered to T1-weighted MRI space, and T1-weighted images were then linearly and non-linearly registered to the Montreal Neurological Institute (MNI) reference space. PET images underwent skull and meninges stripping and were subsequently non-linearly registered to the MNI space using transformations derived from the T1-weighted image to MNI space and PET image to T1-weighted image space. T1-weighted MR images were parcellated into 101 regions using the Desikan-Killiany atlas [26]. No partial volume corrections were applied during the analysis.

### 2.5 Amyloid PET reading and quantification

Amyloid PET images were visually interpreted by assessing regions of interest, including the lateral temporal cortex, frontal cortex, posterior cingulate and precuneus, and inferolateral parietal cortex. Positivity in any of these regions was considered sufficient for an overall positive scan. The final assessment was recorded as a dichotomous classification (positive/negative) based on visual rating, with consensus reached between two nuclear medicine physicians (SH and SYP, both with 14 years of clinical experience in nuclear medicine). Quantitative Standardized uptake value ratio (SUVR) values were used as a supplementary measure to assist visual rating, referring to a predefined global SUVR cutoff of 0.62. The pons was used as the reference region for SUVR calculation in [^18^F]-flutemetamol PET/CT scan studies.

Global [^18^F]-flutemetamol SUVR values were calculated as the average of regional cortical SUVRs from the frontal, superior parietal, lateral temporal, anterior and posterior cingulate cortex, and precuneus, weighted by region size [27]. These global SUVR values were not only used to support the visual assessment but also served as the primary quantitative parameter for the main statistical analyses.

### 2.6 Tau PET reading and quantification

Tau pathology was assessed using Braak staging in the quantitative analysis of [¹⁸F]-flortaucipir PET/CT images. Staging was determined based on topographical involvement of specific brain regions [28]. Braak stage I was defined by tau accumulation in the entorhinal cortex. Braak stages III/IV encompassed regions including the parahippocampal and fusiform gyri, lingual gyrus, amygdala, inferior and middle temporal gyri, temporal pole, thalamus, caudal and rostral anterior cingulate, isthmus and posterior cingulate cortices, and the insula. Braak stages V/VI included more extensive neocortical regions such as the frontal, parietal, and occipital lobes; transverse and superior temporal gyri; precuneus; superior temporal sulcus banks; nucleus accumbens; caudate nucleus; putamen; motor and somatosensory cortices (precentral, postcentral, and paracentral gyri); cuneus; and pericalcarine cortex [29]. Braak stage II, which includes the hippocampus, was excluded from the analysis to avoid potential bias introduced by known off-target binding of [¹⁸F]-flortaucipir in this region [30].

PET images were reviewed following guidelines consistent with regulatory approvals from the FDA and EMA for clinical interpretation. Two experienced nuclear medicine physicians (SH and SYP, each with 14 years of experience) performed visual assessments based on anatomical Braak staging. Quantitative evaluation using SUVR values supported the visual interpretations, with a predefined SUVR threshold of 1.3 used as a reference [31]. In cases of discrepancy between visual interpretation and quantitative analysis, raters re-evaluated the images to reach a consensus decision.

A standardized single-threshold approach was employed to harmonize visual and quantitative findings, aiming to improve clinical consistency in tauopathy classification. This methodology has been validated in prior studies as a reliable predictor of cognitive deterioration throughout the clinical continuum of neurodegenerative disease [28]. Braak stage positivity was defined hierarchically: Braak I positivity indicated tau presence limited to the Braak I ROI; Braak III/IV positivity required additional involvement of the III/IV ROIs; and Braak V/VI positivity was assigned when all three regions (I, III/IV, and V/VI) showed tau accumulation. Participants without tau positivity in any of these ROIs were categorized as Braak stage 0.

The temporal meta-ROI used for quantitative analysis included the entorhinal cortex, amygdala, parahippocampal gyrus, fusiform gyrus, and the inferior and middle temporal gyri [32]. The SUVR for [^18^F]-flortaucipir was calculated using the cerebellar cortex as the reference region to ensure consistency in measurements [28,29].

In the present study, the main analysis utilized the meta-ROI SUVR, as it is a widely used and reliable quantitative marker that accurately reflects tau pathology [33]. Moreover, this approach increased the model degrees of freedom and facilitated a clearer interpretation of interaction effects between variables by using a continuous variable (meta-ROI SUVR) rather than a categorical variable (Braak stages) [34,35].

Although generalized linear models (GLMs) are robust to non-normality of independent variables, the nominal nature of Braak staging and the substantial imbalance in group sizes limited its applicability in the primary analyses. Therefore, we employed the continuous meta-ROI SUVR variable to maximize statistical power and ensure a more stable estimation of interaction effects.

### 2.7 Neuropsychological assessment and other clinical measures

All participants underwent cognitive assessments using the Korean version of the CERAD-K battery [36]. The assessment battery included Korean versions of the following tests: Verbal Fluency (VF), the 15-item Boston Naming Test, and the Korean Mini-Mental State Examination (MMSE-K). Additional cognitive measures included word list memory (WLM), recall, recognition, constructional praxis, and recall (CR). A comprehensive CERAD-K score was calculated by aggregating scores from all tests, excluding MMSE-K and CR [37]. In addition, we also collected data on apolipoprotein E (*APOE*) genotypes, which may act as confounding factors in the depression of older adults [38]. *APOE* genotyping was performed using DNA extracted from participants’ peripheral blood. Participants were then classified based on the presence of the *APOE* ε4 allele, with those carrying at least one ε4 allele designated as *APOE* ε4 carriers, while those without any ε4 alleles were classified as non-carriers.

### 2.8 Depression scales

In this study, depressive symptoms were assessed using three well-validated clinical rating scales: the Cornell Scale for Depression in Dementia (CSDD), the Hamilton Depression Rating Scale (HAM-D), and the Geriatric Depression Scale-Short Version (GDS-SV). Korean-translated and culturally adapted versions of these scales were used, all of which have been validated in previous studies.

The CSDD is a clinician-rated scale specifically developed to assess depressive symptoms in individuals with cognitive impairment or dementia. It consists of 19 items evaluating mood-related signs, behavioral disturbance, physical signs, cyclic functions, and ideational disturbance, with input obtained from both the patient and an informant. The Korean version of the CSDD has demonstrated good reliability and validity in older adults with cognitive decline, with internal consistency (Cronbach’s α) reported to be 0.92 and test-retest reliability of 0.91 [39].

The HAM-D is one of the most widely used clinician-administered depression rating scales and serves as a benchmark for assessing the severity of depressive symptoms, particularly in clinical and research settings. The 17-item version was employed in this study. The Korean version of the HAM-D has shown high internal consistency (Cronbach’s α = 0.89) and inter-rater reliability (*r* = 0.94) in patients with depressive disorders [40].

The GDS-SV is a self-report measure designed for older adults, consisting of 15 yes/no questions. It is useful for screening depression in elderly populations, including those with mild cognitive impairment. The Korean version of the GDS-SV has been validated in community-dwelling older adults, showing good reliability (Cronbach’s α = 0.88) and concurrent validity with other depression scales [41].

These three complementary tools allowed for a comprehensive assessment of depressive symptoms across different cognitive statuses in our cohort, with both informant-based and self-report perspectives.

### 2.9 Statistical analysis

Statistical analyses were conducted using R software (version 4.3.2) and jamovi (version 2.6.26) (https://www.jamovi.org). The main analysis included 24 NC, 54 patients with MCI, and 25 patients with amyloid-positive dementia, all of whom had complete data, including tau PET imaging (Supplementary Fig. 1). Analysis of variance (ANOVA) was used to evaluate group differences.

To further investigate the distribution of plasma MDS-OAβ levels and global amyloid-PET SUVRs according to tau pathology burden, participants were stratified into quartiles based on the tau PET meta-ROI SUVR. The mean ± standard deviation (SD) of plasma MDS-OAβ levels and amyloid plaque burden (measured by global amyloid PET SUVR) were calculated for each tau quartile. In addition, the distribution of these biomarkers was similarly analyzed based on Braak stage classifications (Supplementary Fig. 2).

GLMs were used to examine the interaction effects between plasma MDS-OAβ levels and tau PET SUVR (meta-ROI) on depressive symptom scores, including the CSDD, HAM-D, and GDS-SV. All models were adjusted for age, sex, *APOE* ε4 carrier status, and global CDR score. A parallel analysis was conducted replacing plasma MDS-OAβ with global amyloid-PET SUVR. Linearity assumptions were assessed, and quadratic terms were incorporated in cases where non-linearity was detected. To control for multiple comparisons across models involving distinct depression scales and interaction terms, *p*-values were adjusted using the Benjamini–Hochberg false discovery rate (FDR) correction.

To aid interpretation of interaction effects, simple effect analyses were performed using Type III ANOVA at predefined values of tau PET SUVR (−1 SD, mean, +1 SD), as well as Johnson–Neyman procedures to identify regions along the tau continuum where the association between amyloid pathology and depressive symptoms was statistically significant. As these procedures were intended for interpretative support rather than confirmatory inference, no additional multiple comparison correction was applied. Statistical significance was set at a two-tailed α level of 0.05.

To address concerns regarding diagnostic heterogeneity, supplementary GLMs and stratified simple slope analyses were also conducted after excluding participants with dementia (CDR = 1, *n* = 25). These analyses followed the same covariate adjustment and modeling procedures described above. Both unadjusted and FDR-adjusted *p*-values were reported to ensure transparency.

In addition, to examine the main effects of each biomarker on depressive symptoms, we conducted univariate linear regression analyses with plasma MDS-OAβ, tau PET SUVR, and global Aβ-PET SUVR as predictors and depression scores (CSDD, HAM-D, GDS-SV) as dependent variables. These analyses were unadjusted and intended to contextualize the presence or absence of direct effects. Results of these univariate regressions are presented in Supplementary Table S1 and illustrated in Supplementary Fig. S3.

Finally, to further evaluate whether the observed interaction between plasma MDS-OAβ and tau PET SUVR was attributable to a specific amyloid subgroup, additional stratified analyses were conducted in individuals classified as Aβ-PET positive (*n* = 64) and Aβ-PET negative (*n* = 39) subgroups. GLMs were re-estimated using the same covariate structure and outcome variables (CSDD, HAM-D, and GDS-SV) within these subgroups.

## 3 Results

### 3.1 Characteristics of participants

There were significant differences among NC, those with MCI, and amyloid-positive dementia patients in terms of *APOE* ε4 carrier status, global amyloid PET SUVR, amyloid positivity, and meta-ROI tau PET SUVR. As expected, significant group differences were also observed in CERAD total scores and MMSE scores (Table 1). However, there were no significant differences across diagnostic groups in age, sex, years of education, or MDS-OAβ levels. Notably, depression scores that were measured by all three scales did not differ significantly between diagnostic groups.

**Table 1:** Baseline demographic and clinical characteristics of the study participants.

	NC (*N* = 24)	MCI (*N* = 54)	Aβ‑PET (+) dementia (*N* = 25)	*P* value
Age (mean ± SD, years)	73.0 (7.8)	75.1 (7.0)	74.8 (7.6)	0.486
Sex (female, %)	17 (70.8 %)	40 (74.1 %)	18 (72.0 %)	0.952
Years of education (mean ± SD)	9.7 (5.6)	10.4 (4.6)	11.1 (4.7)	0.618
*APOE* ε4 carrier status (carrier, %)	7 (29.2 %)	14 (25.9 %)	18 (72.0 %)	< 0.001
CSDD (mean ± SD)	6.6 (6.7)	6.7 (4.6)	6.0 (6.3)	0.871
HAM-D (mean ± SD)	8.5 (7.0)	8.8 (4.8)	7.2 (5.0)	0.474
GDS-SV (mean ± SD)	9.6 (7.7)	10.8 (5.5)	8.2 (6.0)	0.230
Plasma MDS-OAβ level (mean ± SD, ng/ml)	0.58 (0.21)	0.62 (0.30)	0.63 (0.22)	0.760
Plasma MDS-OAβ risk (n, %)				0.367
Low	19 (79.2 %)	36 (66.7 %)	16 (64.0 %)	
Intermediate	4 (16.7 %)	11 (20.4 %)	8 (32.0 %)	
High	1 (4.2 %)	7 (13.0 %)	1 (4.0 %)	
Global [^18^F] Flutemetamol SUVR_PONS_ (mean ± SD)	0.58 (0.21)	0.68 (0.21)	0.85 (0.09)	< 0.001
[^18^F] Flutemetamol deposition (positivity, %)	7 (29.2 %)	32 (59.3 %)	25 (100.0 %)	< 0.001
Global [^18^F] Flortaucipir SUVR_CEREBELLUM_ (mean ± SD)	1.10 (0.07)	1.20 (0.18)	1.48 (0.20)	< 0.001
Braak I	1.16 (0.20)	1.51 (0.40)	1.78 (0.24)	< 0.001
Braak III/IV	1.15 (0.09)	1.30 (0.24)	1.63 (0.22)	< 0.001
Braak V/VI	1.07 (0.07)	1.16 (0.17)	1.43 (0.21)	< 0.001
Temporal meta-ROI	1.19 (0.14)	1.44 (0.34)	1.92 (0.33)	< 0.001
Braak stage (n, %)				< 0.001
Braak 0	19 (79.2 %)	27 (50.0 %)	2 (8.0 %)	< 0.001
Braak I	4 (16.7 %)	7 (13.0 %)	0 (0.0 %)	< 0.001
Braak III/IV	1 (4.2 %)	12 (22.2 %)	6 (24.0 %)	< 0.001
Braak V/VI	0 (0.0 %)	8 (14.8 %)	17 (68.0 %)	< 0.001
CERAD-K Battery (mean ± SD)				< 0.001
VF	14.9 (4.3)	10.6 (4.1)	6.6 (3.0)	< 0.001
BNT	12.3 (2.0)	10.2 (2.8)	7.6 (3.2)	< 0.001
MMSE	27.0 (1.9)	23.0 (3.8)	17.4 (4.1)	< 0.001
WLM	18.5 (3.1)	12.5 (3.3)	8.6 (2.9)	< 0.001
CP	10.2 (0.9)	9.6 (1.4)	8.1 (2.4)	< 0.001
WLR	6.3 (1.3)	2.6 (1.7)	0.4 (0.7)	< 0.001
WLRc	9.2 (0.9)	6.4 (2.6)	1.7 (1.6)	< 0.001
CR	6.5 (2.6)	2.5 (2.4)	0.3 (1.1)	< 0.001
CERAD total score	71.2 (8.6)	51.8 (11.4)	32.9 (8.0)	< 0.001

**Note.** Data are presented as mean ± standard deviation (SD) for continuous variables and counts (percentages) for categorical variables. Group comparisons were conducted using ANOVA for continuous variables and chi-square tests for categorical variables. **Abbreviations.** NC, cognitively normal; MCI, mild cognitive impairment; CDR, Clinical Dementia Rating; CSDD, Cornell Scale for Depression in Dementia; HAM-D, Hamilton Depression Rating Scale; GDS-SV, a short version Geriatric Depression Scale; MDS-OAβ, Multimer Detection System–oligomeric amyloid-β; SUVR_PONS_, standardized uptake value ratio of [^18^F] Flutemetamol, using the pons as a reference region; SUVR_CEREBELLUM_, standardized uptake value ratio of [^18^F] Flortaucipir, using the cerebellar cortex as a reference region; CERAD-K, Korean version of Consortium to Establish a Registry for Alzheimer’s Disease; VF, verbal fluency; BNT, Boston Naming Test; MMSE, the Korean version of the Mini-Mental Status Examination; WLM, Word List Memory; CP, Constructional Praxis; WLR, Word List Recall; WLRc, Word List Recognition; CERAD total score, composite score summing scores of the CERAD-K VF, BNT, WLM, CP, WLR, and WLRc domains.

### 3.2 Amyloid beta distribution by tau burden

When tau PET SUVR was divided into quartiles, MDS-OAβ levels showed an increasing trend from the 1st to the 3rd quartile, followed by a decrease in the 4th quartile. In contrast, amyloid plaque burden measured by amyloid PET exhibited a continuous increase across all four tau quartiles. In addition, a similar pattern was observed when examining MDS-OAβ and global amyloid PET SUVR values across different Braak stages (Supplementary Fig. 2).

### 3.3 Interaction between amyloid markers and tau on depression

Significant negative interactions were observed between plasma MDS-OAβ and tau PET SUVR on all three depression scales: CSDD (β = −17.418, 95 % CI: −29.194 to −5.641, FDR-adjusted *p* = 0.036), HAM-D (β = −18.664, 95 % CI: −29.800 to −7.528, FDR-adjusted *p* = 0.018), and GDS-SV (β = −18.185, 95 % CI: −31.162 to −5.207, FDR-adjusted *p* = 0.036) (Table 2). In contrast, no significant interaction effects were observed between global Aβ-PET SUVR and tau PET SUVR on any depression measure after FDR correction.

**Table 2:** Interaction effects between amyloid markers (plasma or PET) and tau PET SUVR on depressive symptom scores.

Depression scale	Amyloid Markers	Independent variable	Estimate (β)	95 % CI	FDR-adj. *P*-value
CSDD	OAβ	Amyloid	0.121	(−3.802, 4.043)	0.952
		Tau	−1.234	(−4.720, 2.253)	0.625
		Amyloid × Tau	−17.418	(−29.194, −5.641)	0.036*
	PET	Amyloid	7.340	(−0.347, 15.028)	0.183
		Tau	−4.792	(−9.285, −0.299)	0.167
		Amyloid × Tau	22.395	(−0.329, 45.118)	0.183
HAM-D	OAβ	Amyloid	1.900	(−1.810, 5.6094)	0.515
		Tau	−1.485	(−4.782, 1.8125)	0.525
		Amyloid × Tau	−18.664	(−29.800, −7.528)	0.018*
	PET	Amyloid	3.387	(−4.153, 10.927)	0.525
		Tau	−3.142	(−7.549, 1.265)	0.292
		Amyloid × Tau	7.306	(−14.982, 29.594)	0.625
GDS-SV	OAβ	Amyloid	0.851	(−3.472, 5.174)	0.741
		Tau	−0.948	(−4.791, 2.894)	0.708
		Amyloid × Tau	−18.185	(−31.162, −5.207)	0.036*
	PET	Amyloid	6.171	(−2.358, 14.700)	0.292
		Tau	−3.973	(−8.958, 1.012)	0.292
		Amyloid × Tau	18.818	(−6.393, 44.029)	0.292

**Note.** GLMs were used to examine the interaction between amyloid markers (MDS-OAβ or global Aβ-PET SUVR) and tau PET SUVR (meta-ROI) on depressive symptom scores measured using the CSDD, HAM-D, and GDS-SV. Covariates included age, sex, *APOE* ε4 carrier status, and global CDR score. *P*-values were adjusted for multiple comparisons using the Benjamini–Hochberg false discovery rate (FDR) correction. *P < 0.05. **Abbreviations.** Same as in Table 1, except: CI, confidence interval; GLM, generalized linear model; FDR, false discovery rate.

To address potential diagnostic heterogeneity, supplementary analyses were conducted after excluding participants with dementia (*n* = 25). The interaction effects between plasma MDS-OAβ and tau PET SUVR on depressive symptoms remained in the same negative direction across all three scales (CSDD: β = –6.554, 95 % CI: –21.084 to 7.975, FDR-adjusted *p* = 0.460; HAM-D: β = –10.011, 95 % CI: –24.681 to 4.660, FDR-adjusted *p* = 0.272; GDS-SV: β = –8.952, 95 % CI: –25.858 to 7.955, FDR-adjusted *p* = 0.401), albeit with reduced statistical significance due to smaller sample size (Supplementary Table S2). Interaction effects between Aβ-PET SUVR and tau remained nonsignificant.

### 3.4 Simple effects of amyloid markers by tau PET SUVR levels

**Fig. 1:**
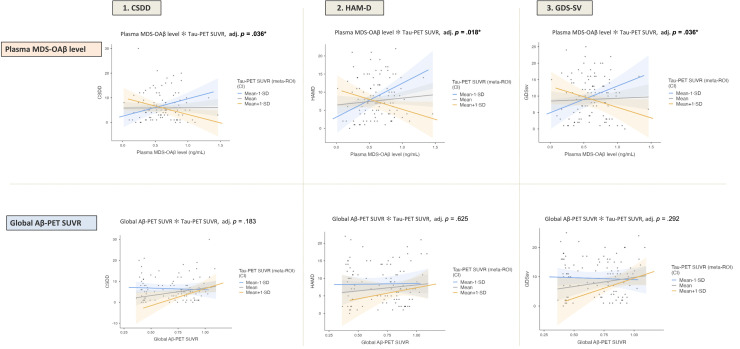
Differential associations between amyloid biomarkers and depressive symptoms across tau pathology levels. Note. General linear models (GLMs) were constructed to test interaction effects between amyloid markers (plasma MDS-OAβ and global Aβ-PET SUVR) and tau PET SUVR (meta-ROI) on depression severity measured by CSDD, HAM-D, and GDS-SV. All models were adjusted for age, sex, *APOE* ε4 carrier status, and global CDR score. *P*-values were adjusted for multiple comparisons using the Benjamini–Hochberg false discovery rate (FDR) correction. *P < 0.05. Abbreviations. Same as in Table 1.

For CSDD, among individuals with low tau burden (Tau −1 SD), higher MDS-OAβ levels were significantly associated with increased depression scores (β = 7.070, 95 % CI: 0.954 to 13.187, *p* = 0.024). In contrast, in the high tau group (Tau +1 SD), higher MDS-OAβ levels were significantly associated with decreased depression scores (β = −6.829, 95 % CI: −13.111 to −0.547, *p* = 0.033). A similar trend was observed when depression was measured by HAM-D (Tau −1 SD: β = 9.350, 95 % CI: 3.560 to 15.131, *p* = 0.002; Tau +1 SD: β = −5.550, 95 % CI: −11.490 to 0.394, *p* = 0.067) and GDS-SV (Tau −1 SD: β = 8.106, 95 % CI: 1.370 to 14.847, *p* = 0.019; Tau +1 SD: β = −6.405, 95 % CI: −13.330 to 0.518, *p* = 0.069) (Table 3 and Fig. 1).

**Table 3:** Simple effect analyses examining the association between amyloid markers and depressive symptom scores stratified by tau PET SUVR.

Depression scale	Tau SUVR level	Amyloid marker	Estimate (β)	95 % CI	*P*-value
CSDD	−1 SD	OAβ	7.070	(0.954, 13.187)	0.024*
		PET	−1.600	(−9.644, 6.450)	0.695
	Mean	OAβ	0.121	(−3.853, 4.094)	0.952
		PET	7.340	(−0.446, 15.130)	0.064
	+1SD	OAβ	−6.829	(−13.111, −0.547)	0.033*
		PET	16.280	(1.271, 31.280)	0.034*
HAM-D	−1 SD	OAβ	9.350	(3.560, 15.131)	**0.002** **
		PET	0.472	(−7.420, 8.370)	0.906
	Mean	OAβ	1.900	(−1.860, 5.657)	0.318
		PET	3.387	(−4.250, 11.020)	0.381
	+1SD	OAβ	−5.550	(−11.490, 0.394)	0.067
		PET	6.303	(−8.410, 21.020)	0.397
GDS-SV	−1 SD	OAβ	8.106	(1.370, 14.847)	0.019*
		PET	−1.340	(−10.270, 7.590)	0.767
	Mean	OAβ	0.851	(−3.530, 5.229)	0.701
		PET	6.170	(−2.470, 14.810)	0.159
	+1SD	OAβ	−6.405	(−13.330, 0.518)	0.069
		PET	13.680	(−2.970, 30.330)	0.106

**Note.** Simple effect analyses were conducted to clarify the interaction between amyloid markers (MDS-OAβ or global Aβ-PET SUVR) and tau PET SUVR on depressive symptoms. Associations were evaluated at three stratified levels of tau PET SUVR (−1 SD, mean, and +1 SD) using Type III sum-of-squares ANOVA. Statistical significance was assessed using chi-square tests (two-tailed *p* < 0.05). All models were adjusted for age, sex, *APOE* ε4 carrier status, and global CDR score. *p < 0.05, ^⁎⁎^*p* < 0.01. **Abbreviations.** Same as in Table 1, except: CI, confidence interval.

With regards to amyloid plaque burden, no significant association with CSDD scores was found in the low tau group (β = −1.600, 95 % CI: −9.644 to 6.450, *p* = 0.695). However, in the high tau group, greater plaque burden was significantly associated with higher depression scores (β = 7.560, 95 % CI: 1.271 to 31.280, *p* = 0.034). No significant associations were observed between amyloid plaque and depression when using HAM-D (Tau −1 SD: β = 0.472, 95 % CI: −7.420 to 8.370, *p* = 0.906; Tau +1 SD: β = 6.303, 95 % CI: −8.410 to 21.020, *p* = 0.397) or GDS-SV (Tau −1 SD: β = −1.340, 95 % CI: −10.270 to 7.590, *p* = 0.767; Tau +1 SD: β = 13.680, 95 % CI: −2.970 to 30.330, *p* = 0.106) (Table 3 and Fig. 1).

To examine whether the observed associations were influenced by the inclusion of participants with dementia, simple slope analyses were repeated after excluding dementia cases (*n* = 25). The direction and magnitude of the associations between plasma MDS-OAβ and depressive symptom scores remained similar across stratified tau PET SUVR levels. In the low tau group, MDS-OAβ was positively associated with HAM-D (β = 9.010, *p* = 0.010), and this association was reduced in the high tau group (β = 2.720, *p* = 0.370). For Aβ-PET SUVR, the estimated effect size on CSDD was greater in the high tau group (β = 8.337, *p* = 0.201) than in the low tau group (β = –0.857, *p* = 0.856). Full results are reported in Supplementary Table S3.

### 3.5 Cutoff points of meta-ROI tau SUVR where the direction of association between amyloid beta and depression scores changed

**Fig. 2:**
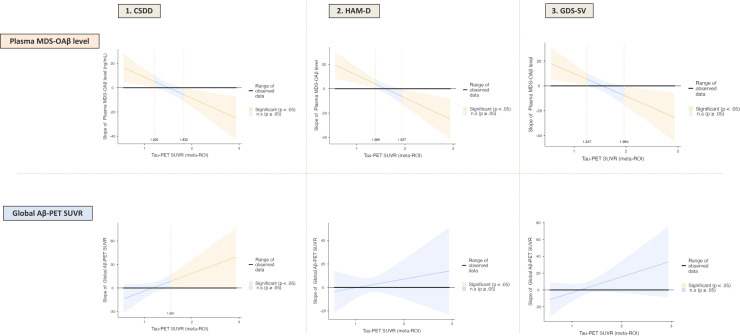
Tau PET SUVR thresholds moderating the association between amyloid biomarkers and depressive symptom scores. Note. Johnson–Neyman plots illustrate tau PET SUVR levels at which the association between amyloid biomarkers (plasma MDS-OAβ or global Aβ-PET SUVR) and depression scores (CSDD, HAM-D, GDS-SV) reached statistical significance. Shaded regions denote confidence bands, and colored segments indicate significant regions (*P* < 0.05). All models were adjusted for age, sex, *APOE* ε4 carrier status, and global CDR score. Abbreviations. Same as in Table 1.

According to the Johnson-Neyman plots, the meta-ROI tau PET SUVR thresholds at which the positive association between MDS-OAβ levels and depression scores ceased to be significant were 1.205 for CSDD, 1.389 for HAM-D, and 1.247 for GDS-SV. The cutoff points at which the reverse (negative) association became statistically significant were 1.832, 1.937, and 1.964, respectively. In the case of amyloid plaque burden, a significant positive association with depression was observed only for CSDD, and only when tau PET SUVR exceeded 1.551 (Fig. 2).

### 3.6 Stratified analysis by Aβ-PET status

To evaluate whether the interaction between plasma MDS-OAβ and tau PET SUVR was preserved among individuals with confirmed amyloid pathology, stratified analyses were conducted within the Aβ-PET positive subgroup (*n* = 64). As summarized in Supplementary Table S4, the interaction remained statistically significant for the CSDD (β = –18.856, 95 % CI: –36.432 to –1.281, *p* = 0.035) and HAM-D (β = –17.354, 95 % CI: –33.565 to –1.143, *p* = 0.036), and marginally significant for the GDS-SV (β = –18.134, 95 % CI: –36.580 to 0.312, *p* = 0.054).

Additionally, simple effect analyses were conducted at stratified levels of tau PET SUVR (–1 SD, mean, +1 SD) within the Aβ-PET positive subgroup. Higher plasma MDS-OAβ levels were associated with lower depressive symptom scores at high tau PET SUVR (+1 SD) across all depression measures: CSDD (β = –11.18, 95 % CI: –20.70 to –1.66, *p* = 0.022), HAM-D (β = –8.91, 95 % CI: –17.69 to –0.13, *p* = 0.047), and GDS-SV (β = –10.64, 95 % CI: –20.63 to –0.65, *p* = 0.037). No significant associations were observed at low or mean tau PET SUVR levels. Full results are provided in Supplementary Table S5.

To complement these findings, analyses were also conducted within the Aβ-PET negative subgroup (*n* = 39). In this group, the interaction between plasma MDS-OAβ and tau PET SUVR was not statistically significant for any depression measures (all *p* > 0.3, Supplementary Table S4). Simple effect analyses were additionally performed for completeness, and the full results are summarized in Supplementary Table S5.

## 4 Discussion

In the current study, only soluble Aβ (MDS-OAβ), but not plaque Aβ measured by amyloid PET, showed significant interactions with tau pathology in predicting depressive symptoms. Specifically, significant negative interactions were observed between MDS-OAβ and tau PET SUVR across all three depression measures, whereas no significant interaction was found for Aβ-PET SUVR. This dissociation highlights the distinct temporal and mechanistic roles of soluble amyloid-β oligomers and insoluble amyloid plaques in neuropsychiatric outcomes. Among participants with low tau SUVR, higher MDS-OAβ levels were positively associated with depressive symptoms, whereas this association was reversed in participants with high tau SUVR. In other words, MDS-OAβ and tau SUVR exhibited a significant negative interaction across the tau continuum. Supporting this pattern, stratified analyses restricted to the Aβ-PET positive subgroup demonstrated a significant interaction between plasma MDS-OAβ and tau PET SUVR on depressive symptoms, reinforcing the robustness of this effect in individuals with confirmed amyloid pathology. Notably, in univariate analyses, none of the individual biomarkers were significantly associated with depression scores across the three measures. This underscores the importance of considering tau pathology as a moderator, rather than a direct correlate of depressive symptoms. The combination of non-significant main effects and significant interaction effects supports a model wherein the influence of amyloid pathology on mood symptoms depends on the level of tau burden.

This result may be explained by previous studies suggesting that OAβ levels tend to peak before substantial tau deposition occurs in the brain and gradually decline thereafter [42]. Thus, in participants at an early stage with low tau deposition, neurotoxicity from oligomeric amyloid beta may have contributed to depressive symptoms. Previous animal studies have shown that OAβ contributes to the onset and exacerbation of depressive symptoms. Proposed mechanisms include synaptic dysfunction, neuroinflammation, and region-specific imbalances in monoamines and nerve growth factor within the brain [43,44]. Conversely, in participants at a more advanced stage with high tau deposition, OAβ levels likely declined due to conversion into amyloid plaques [42], and depressive symptoms may have been more directly driven by the increasing tau pathology [11]. Supporting this explanation, ANOVA analysis across tau SUVR level quartiles and Braak stages in the present study showed that while MDS-OAβ levels initially increased with tau levels, they declined in the highest tau quartile. In contrast, amyloid plaque levels (global amyloid PET SUVR) continued to increase linearly (Supplementary Fig. S2).

A minor but noteworthy finding was that, although not consistently across all depression measures, in the high tau group, global amyloid PET SUVR showed a positive association with depression. As previously reported, the formation of amyloid plaques tends to occur in a timeframe similar to tau deposition [42]. Therefore, in the high tau group of this study, it is plausible that depressive symptoms were more directly related to deposited tau rather than the plaques themselves. Nonetheless, the neurotoxicity of the plaques may also have contributed to depressive symptoms. Supporting this, previous studies have reported a significant association between amyloid plaques measured by amyloid PET and depressive symptoms [45,46]. However, studies that examined the relationship between pre-mortem depressive symptoms and postmortem amyloid plaque burden in older adult cohorts have shown mixed evidence [[47], [48], [49]]. In the present study, the effect of amyloid plaques on depressive symptoms was smaller than that of OAβ. Considering the typical sequence of AD pathology that is amyloid accumulation, followed by tau deposition, and eventually widespread neurodegeneration [50], this finding might suggest that depressive symptoms emerging as a prodrome before substantial neurodegeneration in AD might be more strongly driven by OAβ than by amyloid plaque accumulation.

According to the Johnson-Neyman plot, MDS-OAβ was positively associated with depression at tau SUVR levels between 1.2 and 1.4. This corresponds well with previously established thresholds from visual reads of meta-ROI tau SUVR, such as 1.41, or +2 SD above the mean of amyloid-negative controls, which is approximately 1.28 [51]. These findings may indicate that OAβ is more strongly related to depressive symptoms during earlier stages of tau pathology, prior to reaching established thresholds of abnormal tau accumulation. While no clear association was observed when OAβ levels were examined across tau SUVR quartiles, a modest inverted-U pattern emerged when stratifying participants by Braak stage, suggesting stage-dependent dynamics in OAβ burden (Supplementary Fig. S2). In contrast, at higher tau SUVR levels (1.832, 1.937, and 1.964), the association between MDS-OAβ and depressive symptoms became significantly negative. These tau levels are consistent with prior evidence indicating abnormal tau accumulation extending into the temporal neocortex, corresponding to more advanced Braak stages [52]. At these later stages, the association patterns may reflect a shift wherein OAβ levels are reduced due to plaque conversion, and depressive symptoms may be more closely linked to the extent of tau-related neurobiological alterations. This interpretation is further supported by the observed distributional patterns of MDS-OAβ and Aβ-PET SUVR across tau strata in the current study (Supplementary Fig. S2).

Regarding amyloid plaque, a significant positive association with depression was observed only in the CSDD at tau SUVR ≥ 1.551. Previous research suggests that a meta-ROI SUVR in the 1.5 range likely reflects the transition between Braak stages II and III, indicating tau pathology is expanding beyond the mesial temporal lobe to the lateral temporal neocortex [53]. This phase also corresponds to a period of accelerated neurodegeneration, during which amyloid plaque might contribute to depressive symptoms. However, this association was not replicated using other depression measures such as HAM-D or GDS-SV.

This discrepancy may be due to differences in the characteristics of the depression rating scales. The CSDD is considered more reliable for assessing depression in patients with dementia compared to other scales, as it captures a broader range of depression-related features unique to dementia and incorporates both patient and informant input [39,54]. In contrast, the GDS-SV is a self-report measure and may be less reliable in individuals with cognitive impairment [55], while the HAM-D may underreport depressive symptoms in apathetic dementia patients [56,57]. Thus, the observed amyloid plaque effect on depression in the high tau group may have only reached statistical significance when assessed with the CSDD.

Nevertheless, these results should be interpreted with caution in view of several limitations inherent to the study, and they underscore the need for future research. First, due to the cross-sectional design, causal relationships among OAβ, tau, and depressive symptoms cannot be established. Prospective longitudinal studies are required to elucidate the temporal sequence and potential causality underlying these associations. Although we conducted descriptive stratifications of plasma MDS-OAβ and global amyloid-PET SUVR across tau PET quartiles and Braak stages, we acknowledge that the absence of clear monotonic trends in these biomarker distributions may limit the interpretability of visual inspection. Accordingly, the interpretation of our findings has been grounded in model-based statistical analyses rather than descriptive stratification, and the presented interaction effects should be interpreted within this analytical framework. This methodological emphasis is critical given the potential for misinterpretation when relying solely on visual inferences from subgroup stratifications, and future studies employing longitudinal designs and alternative statistical modeling approaches are warranted to validate the observed interaction patterns. Second, although we measured the burden of Aβ oligomers, this study did not include direct assessment of soluble tau species. As tau pathology may contribute to depressive symptoms, the lack of soluble tau data might have led to an underestimation of its impact. Third, the study was conducted within a single-center East Asian cohort, which may restrict the generalizability of the findings due to possible selection bias and known racial and ethnic variations in AD pathophysiology and depression vulnerability. Fourth, while the MDS reflects the propensity for Aβ oligomerization rather than providing absolute quantification, it remains effective in distinguishing oligomers from monomers and in capturing relative changes in oligomer levels [14,58]. Longitudinal studies with extended follow-up are warranted to clarify the progression of AD-related pathologies and their relationship to depression over time.

In conclusion, the present study suggests that MDS-OAβ is significantly associated with the severity of depressive symptoms prior to the substantial accumulation of tau pathology. However, in individuals with advanced tau pathology involving widespread neocortical regions, tau deposition itself or possibly amyloid plaque burden may have a stronger association with depressive symptoms than plasma MDS-OAβ levels. These findings suggest that plasma MDS-OAβ may serve as a potential biomarker for identifying early affective manifestations of AD, prior to the emergence of advanced tau pathology. Furthermore, this biomarker may assist in refining clinical stratification and guiding the design of research protocols targeting early neuropsychiatric phenotypes within the AD continuum.

## Funding

This research was supported by the Basic Science Research Program through the 10.13039/501100003725National Research Foundation of Korea (NRF) funded by the Ministry of Education (2022R1I1A1A01053710); a research fund of Seoul St. Mary’s Hospital, the Catholic University of Korea (ZC23TISI0860); the Culture, Sports, and Tourism R&D Program through the 10.13039/501100006465Korea Creative Content Agency grant funded by the Ministry of Culture, Sports, and Tourism of Korea in 2023 (R2022020030); the Basic Medical Science Facilitation Program through the Catholic Medical Center of the Catholic University of Korea funded by the Catholic Education Foundation; and the Institute of Clinical Medicine Research at Yeouido St. Mary’s Hospital, the 10.13039/501100002648Catholic University of Korea.

## Availability of data and materials

The datasets generated or analyzed during the current study are not publicly available due to the Patient Data Management Protocol of Yeouido Saint Mary’s Hospital but are available from the corresponding author upon reasonable request.

## CRediT authorship contribution statement

**Gihwan Byeon:** Writing – review & editing, Writing – original draft, Visualization, Validation, Software, Resources, Methodology, Investigation, Formal analysis, Conceptualization. **Suhyung Kim:** Validation, Resources, Investigation, Data curation. **Sunghwan Kim:** Validation, Project administration, Methodology, Investigation, Data curation. **Yoo Hyun Um:** Validation, Resources, Project administration, Methodology, Investigation, Data curation. **Sheng-Min Wang:** Validation, Software, Resources, Project administration, Methodology, Investigation, Data curation. **Seunggyun Ha:** Validation, Resources, Methodology, Investigation, Data curation. **Sonya Youngju Park:** Software, Resources, Methodology, Investigation, Data curation. **Yeong Sim Choe:** Resources, Project administration, Methodology, Investigation, Data curation. **Donghyeon Kim:** Validation, Software, Resources, Methodology, Investigation, Data curation. **Hyun Kook Lim:** Supervision, Resources, Project administration, Methodology, Investigation, Funding acquisition, Data curation, Conceptualization. **Chang Uk Lee:** Validation, Resources, Methodology, Investigation, Data curation. **Dong Woo Kang:** Writing – review & editing, Writing – original draft, Visualization, Validation, Supervision, Software, Resources, Project administration, Methodology, Investigation, Funding acquisition, Formal analysis, Data curation, Conceptualization.

## Declaration of competing interest

Hyun Kook Lim was employed by NEUROPHET Inc. The remaining authors declare that the research was conducted in the absence of any commercial or financial relationships that could be construed as potential conflicts of interest. The data processing services provided by NEUROPHET Inc. were used to enhance the quality and analysis of the brain imaging data collected during the study. The authors declare that the research outcomes and conclusions remain unbiased and are not influenced by any commercial interests associated with NEUROPHET Inc.'s products or services.

## Appendix Supplementary materials

Image, application 1
