# Supplementary material for: Differential effects of soluble and plaque amyloid on late-life depression: The moderating role of tau pathology
Source: J Prev Alzheimers Dis. 2025 Aug 5;12(9):100318. doi: 10.1016/j.tjpad.2025.100318 (PMC12501351; doi:10.1016/j.tjpad.2025.100318)
Supplement: Supplementary file 1 [file mmc1.docx]

**Additional File 1: Supplementary Tables, and Figures**

**File format:** .docx
**Title:** Supplementary Materials for "Differential Effects of Soluble and Plaque Amyloid on Late-Life Depression: The Moderating Role of Tau Pathology"
**Description:** This file includes supplementary results, additional subgroup and sensitivity analyses, and supporting figures for the main manuscript. It contains four supplementary tables (S1–S5) and three figures (S1–S3) detailing analytic procedures, model outputs, and exploratory results.

**Table of Contents**

1. Supplementary Tables
   - Table S1. Univariate associations between individual AD biomarkers and depression scales (CSDD, HAM-D, GDS-SV)
   - Table S2. Interaction effects between amyloid markers and tau PET SUVR on depressive symptom scores after excluding participants with dementia
   - Table S3. Simple effects of amyloid markers stratified by tau PET SUVR levels after excluding participants with dementia
   - Table S4. Interaction effects between plasma MDS-OAβ and tau PET SUVR on depressive symptoms within the Aβ-PET positive subgroup
   - Table S5. Simple effect analyses stratified by tau PET SUVR levels within the Aβ-PET positive subgroup
2. Supplementary Figures
   - Figure S1. Flowchart of participant selection and data availability
   - Figure S2. Distribution of amyloid beta across tau PET quartiles and Braak stages
   - Figure S3. Univariate associations between AD core biomarkers and depressive symptom scores

**Supplementary Tables**

**Table S1. Univariate Linear Regression Analyses Between AD Core Biomarkers and Depression Scores**

| Depression scale | AD Core Biomarkers | Estimate (β) | 95% CI | *P*-value |
| --- | --- | --- | --- | --- |
| CSDD | Plasma MDS-OAβ | -0.64 | (-4.748, 3.475) | 0.762 |
|  | Global Aβ PET SUVR | 0.54 | (-4.632, 5.715) | 0.838 |
|  | Tau PET SUVR | -0.72 | (-3.422, 1.986) | 0.604 |
| HAM-D | Plasma MDS-OAβ | 0.98 | (-3.036, 4.994) | 0.634 |
|  | Global Aβ PET SUVR | -0.85 | (-5.905, 4.202) | 0.742 |
|  | Tau PET SUVR | -1.18 | (-3.817, 1.454) | 0.382 |
| GDS-SV | Plasma MDS-OAβ | -0.12 | (-4.735, 4.488) | 0.958 |
|  | Global Aβ PET SUVR | -0.21 | (-6.013, 5.589) | 0.943 |
|  | Tau PET SUVR | -0.82 | (-3.847, 2.216) | 0.599 |

**Note.** Simple linear regressions were conducted to evaluate the individual associations between AD core biomarkers (plasma MDS-OAβ, global Aβ PET SUVR, and tau PET SUVR) and depressive symptom scores (CSDD, HAM-D, GDS-SV) without adjustment for covariates. None of the univariate associations reached statistical significance. **Abbreviations.** Same as in Table 1, except: CI, confidence interval.

**Table S2. Interaction effects between amyloid markers and tau PET SUVR on depressive symptom scores after excluding participants with dementia**

| Depression scale | Amyloid Measures | Independent variable | Estimate (β) | 95% CI | Raw  *P*-value | FDR-adj.  *P*-value |
| --- | --- | --- | --- | --- | --- | --- |
| CSDD | OAβ | Amyloid | 4.483 | (0.189, 8.776) | 0.041 | 0.061 |
|  |  | Tau | -1.897 | (-5.966, 2.172) | 0.361 | 0.432 |
|  |  | Amyloid × Tau | -6.554 | (-21.084, 7.975) | 0.377 | 0.460 |
|  | PET | Amyloid | 3.74 | (-3.542, 11.022) | 0.314 | 0.411 |
|  |  | Tau | -4.777 | (-11.149, 1.594) | 0.142 | 0.284 |
|  |  | Amyloid × Tau | 14.641 | (-11.951, 41.234) | 0.281 | 0.439 |
| HAM-D | OAβ | Amyloid | 5.867 | (1.532, 10.202) | 0.008 | 0.024 |
|  |  | Tau | -1.46 | (-5.569, 2.648) | 0.486 | 0.531 |
|  |  | Amyloid × Tau | -10.011 | (-24.681, 4.66) | 0.181 | 0.272 |
|  | PET | Amyloid | 2.427 | (-5.158, 10.012) | 0.531 | 0.577 |
|  |  | Tau | -2.832 | (-9.469, 3.805) | 0.403 | 0.519 |
|  |  | Amyloid × Tau | 6.623 | (-21.077, 34.323) | 0.639 | 0.720 |
| GDS-SV | OAβ | Amyloid | 4.756 | (-0.24, 9.752) | 0.062 | 0.091 |
|  |  | Tau | -0.018 | (-4.753, 4.717) | 0.994 | 0.994 |
|  |  | Amyloid × Tau | -8.952 | (-25.858, 7.955) | 0.299 | 0.401 |
|  | PET | Amyloid | 2.411 | (-6.062, 10.884) | 0.577 | 0.642 |
|  |  | Tau | -2.726 | (-10.139, 4.688) | 0.471 | 0.588 |
|  |  | Amyloid × Tau | 17.127 | (-13.816, 48.07) | 0.278 | 0.397 |

**Note.** GLMs were used to examine the interaction between amyloid markers (plasma MDS-OAβ or global Aβ-PET SUVR) and tau PET SUVR (meta-ROI) on depressive symptom scores (CSDD, HAM-D, GDS-SV) after excluding dementia participants (N = 25). Covariates included age, sex, *APOE* ε4 carrier status, and global CDR score. Both unadjusted and Benjamini–Hochberg FDR-adjusted p-values are reported. **Abbreviations.** Same as in Table 1 and Table S1, except: GLM, generalized linear model; FDR, false discovery rate.

**Table S3. Simple effect analyses stratified by tau PET SUVR levels after excluding participants with dementia**

| Depression scale | Tau-PET SUVR level | Amyloid  marker | Estimate (β) | 95% CI | *P*-value |
| --- | --- | --- | --- | --- | --- |
| CSDD | -1 SD | OAβ | 6.54 | (-0.217, 13.3) | 0.058 |
|  |  | PET | -0.857 | (-10.23, 8.52) | 0.856 |
|  | Mean | OAβ | 4.48 | (0.114, 8.85) | 0.044 |
|  |  | PET | 3.74 | (-3.67, 11.15) | 0.318 |
|  | +1SD | OAβ | 2.42 | (-3.543, 8.39) | 0.42 |
|  |  | PET | 8.337 | (-4.56, 21.23) | 0.201 |
| HAM-D | -1 SD | OAβ | 9.01 | (2.19, 15.83) | 0.01 |
|  |  | PET | 0.348 | (-9.42, 10.1) | 0.944 |
|  | Mean | OAβ | 5.87 | (1.46, 10.28) | 0.01 |
|  |  | PET | 2.427 | (-5.29, 10.1) | 0.533 |
|  | +1SD | OAβ | 2.72 | (-3.3, 8.75) | 0.37 |
|  |  | PET | 4.507 | (-8.93, 17.9) | 0.506 |
| GDS-SV | -1 SD | OAβ | 7.57 | (-0.297, 15.43) | 0.059 |
|  |  | PET | -2.97 | (-13.88, 7.94) | 0.589 |
|  | Mean | OAβ | 4.76 | (-0.328, 9.84) | 0.066 |
|  |  | PET | 2.41 | (-6.21, 11.03) | 0.579 |
|  | +1SD | OAβ | 1.95 | (-4.998, 8.89) | 0.578 |
|  |  | PET | 7.79 | (-7.22, 22.79) | 0.304 |

**Note.** Simple effect analyses were conducted at stratified levels of tau PET SUVR (−1 SD, mean, +1 SD) to clarify interaction effects between amyloid markers and tau PET SUVR on depressive symptom scores (CSDD, HAM-D, GDS-SV) after excluding dementia participants. Type III sum-of-squares ANOVA was used. Models were adjusted for age, sex, *APOE* ε4 carrier status, and global CDR score. **Abbreviations.** Same as in Table 1 and Table S1.

**Table S4. Interaction effects between plasma MDS-OAβ and tau PET SUVR on depressive symptoms stratified by Aβ-PET status**

(A) Aβ-PET positive subgroup (n = 64)

| Depression scale | Amyloid Measures | Independent variable | Estimate (β) | 95% CI | *P*-value |
| --- | --- | --- | --- | --- | --- |
| CSDD | OAβ | Amyloid | -4.048 | (-9.366, 1.270) | 0.136 |
|  |  | Tau | -0.560 | (-4.939, 3.819) | 0.802 |
|  |  | Amyloid × Tau | -18.856 | (-36.432, -1.281) | 0.035 |
| HAM-D | OAβ | Amyloid | -2.348 | (-7.253, 2.557) | 0.348 |
|  |  | Tau | -0.984 | (-7.253, 3.055) | 0.633 |
|  |  | Amyloid × Tau | -17.354 | (-33.565, -1.143) | 0.036 |
| GDS-SV | OAβ | Amyloid | -3.782 | (-9.364, 1.799) | 0.184 |
|  |  | Tau | -0.033 | (-4.629, 4.562) | 0.989 |
|  |  | Amyloid × Tau | -18.134 | (-36.580, 0.312) | 0.054 |

(B) Aβ-PET negative subgroup (n = 39)

| Depression scale | Amyloid Measures | Independent variable | Estimate (β) | 95% CI | *P*-value |
| --- | --- | --- | --- | --- | --- |
| CSDD | OAβ | Amyloid | 6.687 | (-0.689, 14.063) | 0.076 |
|  |  | Tau | -7.719 | (-24.936, 9.499) | 0.380 |
|  |  | Amyloid × Tau | -3.835 | (-36.427, 28.758) | 0.818 |
| HAM-D | OAβ | Amyloid | 9.839 | (2.695, 16.983) | 0.007 |
|  |  | Tau | -3.774 | (-20.449, 12.902) | 0.657 |
|  |  | Amyloid × Tau | -13.548 | (-45.114, 18.019) | 0.400 |
| GDS-SV | OAβ | Amyloid | 8.571 | (-0.018, 17.160) | 0.050 |
|  |  | Tau | -6.767 | (-26.815, 13.281) | 0.508 |
|  |  | Amyloid × Tau | -6.384 | (-44.335, 31.567) | 0.742 |

**Note.** GLMs were conducted to examine the interaction between plasma MDS-OAβ and tau PET SUVR on depressive symptoms (CSDD, HAM-D, and GDS-SV) within Aβ-PET positive and negative subgroups. All models were adjusted for age, sex, *APOE* ε4 carrier status, and global CDR score. Reported *p*-values are uncorrected due to the exploratory nature of this subgroup analysis. **Abbreviations.** Same as in Table 1 and Table S1.

**Table S5. Simple effect analyses stratified by tau PET SUVR levels stratified by Aβ-PET status**

(A) Aβ-PET positive subgroup (n = 64)

| Depression scale | Tau-PET SUVR level | Amyloid  marker | Estimate (β) | 95% CI | *P*-value |
| --- | --- | --- | --- | --- | --- |
| CSDD | -1 SD | OAβ | 3.08 | (-4.71, 10.87) | 0.432 |
|  | Mean | OAβ | -4.05 | (-9.48, 1.39) | 0.141 |
|  | +1SD | OAβ | -11.18 | (-20.70, -1.66) | 0.022 |
| HAM-D | -1 SD | OAβ | 4.21 | (-2.97, 11.396) | 0.245 |
|  | Mean | OAβ | -2.35 | (-7.36, 2.665) | 0.352 |
|  | +1SD | OAβ | -8.91 | (-17.69, -0.127) | 0.047 |
| GDS-SV | -1 SD | OAβ | 3.07 | (-5.10, 11.247) | 0.455 |
|  | Mean | OAβ | -3.78 | (-9.49, 1.922) | 0.190 |
|  | +1SD | OAβ | -10.64 | (-20.63, -0.645) | 0.037 |

(B) Aβ-PET negative subgroup (n = 39)

| Depression scale | Tau-PET SUVR level | Amyloid  marker | Estimate (β) | 95% CI | *P*-value |
| --- | --- | --- | --- | --- | --- |
| CSDD | -1 SD | OAβ | 7.35 | (0.148, 17.5) | 0.148 |
|  | Mean | OAβ | 6.69 | (0.085, 14.4) | 0.085 |
|  | +1SD | OAβ | 6.02 | (0.191, 15.2) | 0.191 |
| HAM-D | -1 SD | OAβ | 12.18 | (2.39, 22.0) | 0.016 |
|  | Mean | OAβ | 9.84 | (2.41, 17.3) | 0.011 |
|  | +1SD | OAβ | 7.50 | (-1.41, 16.4) | 0.096 |
| GDS-SV | -1 SD | OAβ | 9.68 | (-2.096, 21.4) | 0.104 |
|  | Mean | OAβ | 8.57 | (-0.366, 17.5) | 0.060 |
|  | +1SD | OAβ | 7.47 | (-3.236, 18.2) | 0.165 |

**Note.** Simple effect analyses were conducted at stratified levels of tau PET SUVR (−1 SD, mean, +1 SD) to clarify interaction effects between amyloid markers and tau PET SUVR on depressive symptom scores (CSDD, HAM-D, GDS-SV) within Aβ-PET positive and negative subgroups. Type III sum-of-squares ANOVA was used. Models were adjusted for age, sex, *APOE* ε4 carrier status, and global CDR score. **Abbreviations.** Same as in Table 1 and Table S1.

**Supplementary Figures**

**Figure S1. Flowchart of Participant Selection and Data Availability**

**
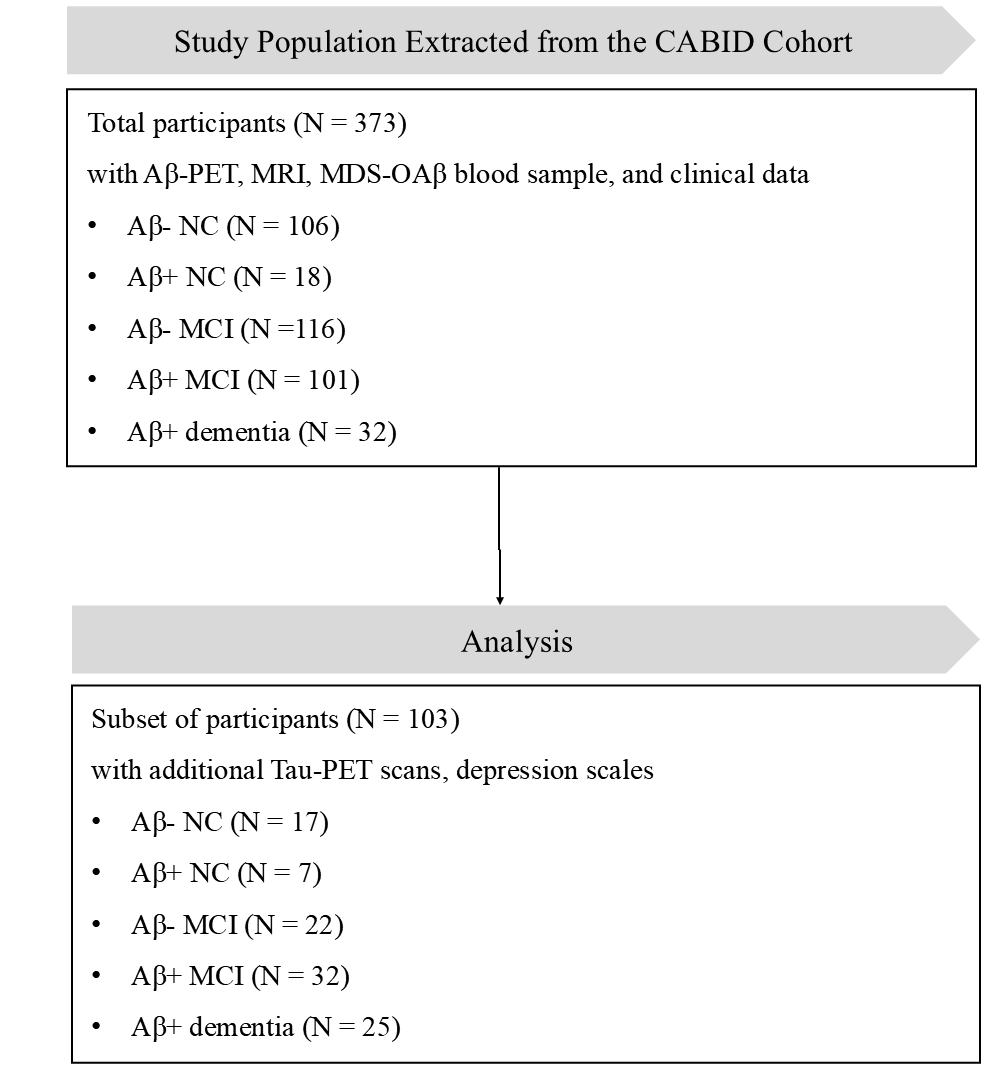
**

**Note. Flow diagram illustrating the selection process of study participants from the CABID cohort. The initial sample included 373 individuals with Aβ-PET, MRI, plasma MDS-OAβ data, and clinical evaluations. A final analytic subset of 103 participants with additional tau-PET imaging and depression scale assessments was used in the main analysis. Diagnostic subgroups are shown according to amyloid status and clinical diagnosis. Abbreviations. Aβ, amyloid-beta; NC, normal cognition; MCI, mild cognitive impairment.**

**Figure S2. Distribution of amyloid beta according to tau burden (Meta-ROI tau SUVR and Braak stage)**


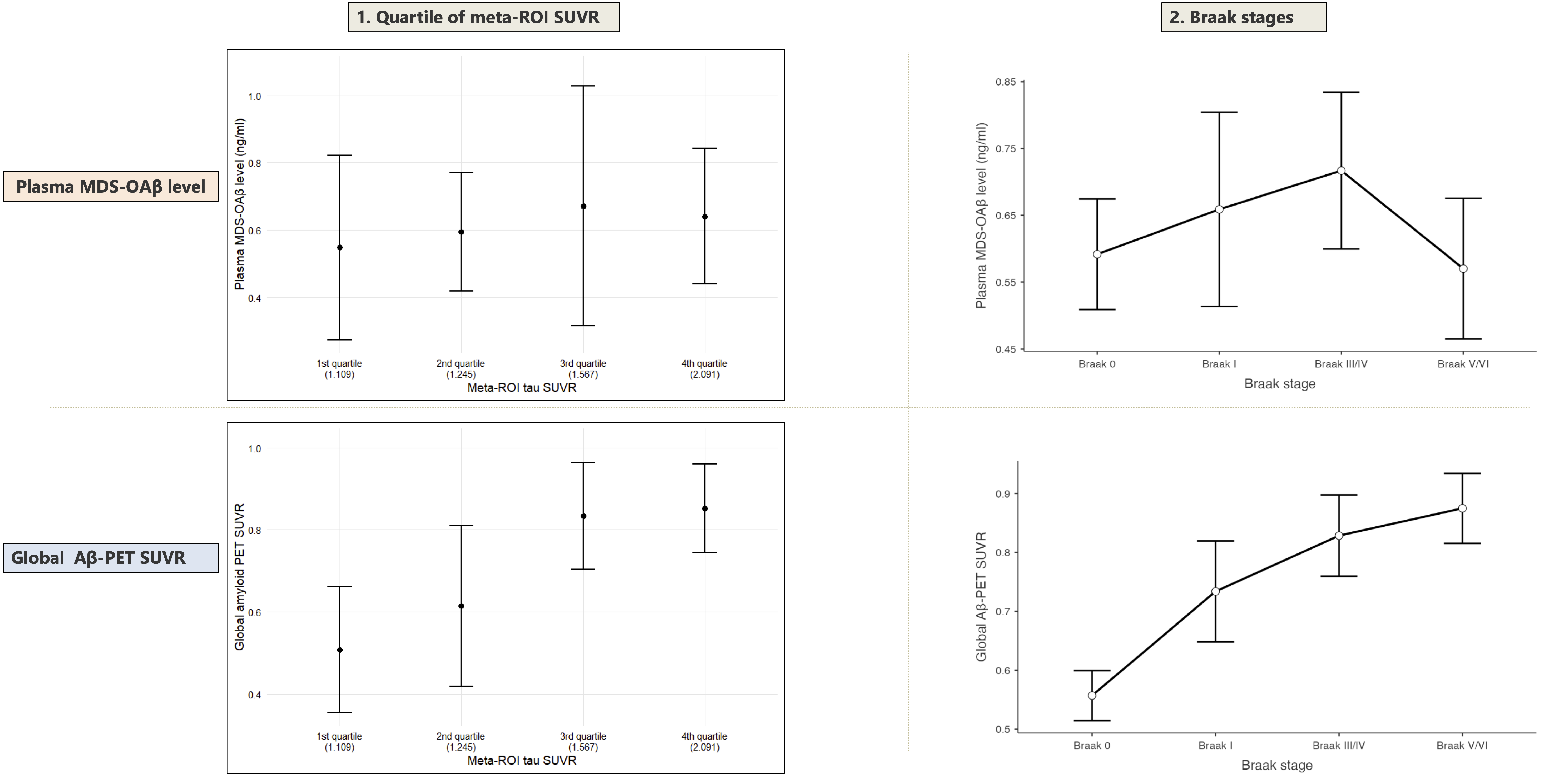


**Note.** Group-level distribution of plasma MDS-OAβ levels and global amyloid-PET SUVR across (1) tau PET SUVR quartiles (left panels) and (2) Braak stages (right panels). Data points represent group means with error bars indicating standard deviations. **Abbreviations.** SUVR, standardized uptake value ratio; Aβ, amyloid-beta; MDS-OAβ, Multimer Detection System-oligomeric amyloid-beta; PET, positron emission tomography.

**Figure S3. Scatter plots showing univariate associations between AD core biomarkers and depressive symptom scores**


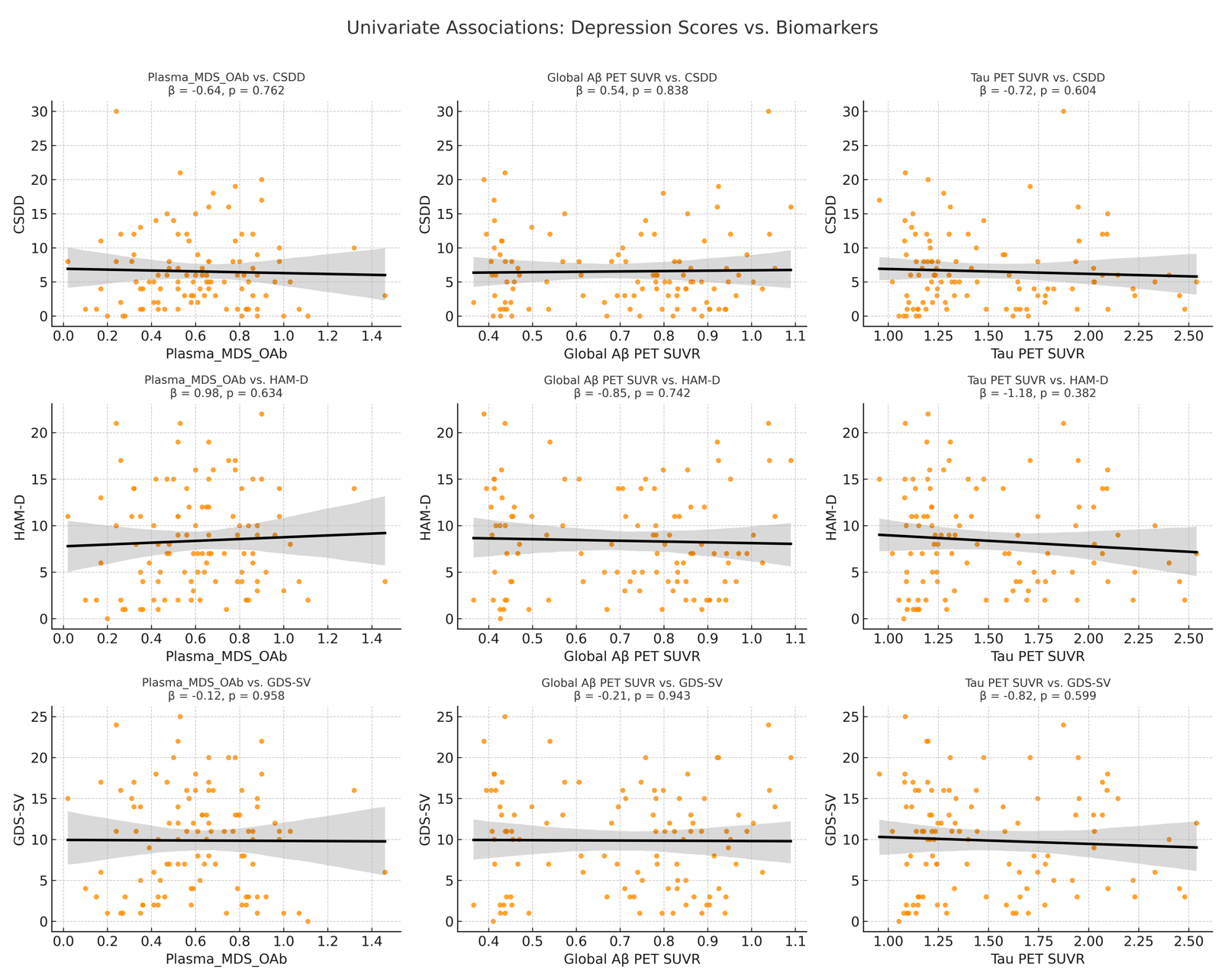


**Note.** Univariate associations between plasma MDS-OAβ, global Aβ PET SUVR, and tau PET SUVR and depressive symptom scores (CSDD, HAM-D, GDS-SV) were visualized using simple linear regression. Each subplot includes the fitted regression line (black) with 95% confidence intervals (gray shaded band). Regression coefficients (β) and *p*-values are presented in the subplot titles. None of the associations were statistically significant. **Abbreviations.** Same as in Table 1, except: CI, confidence interval.
